# Privacy Relevance and Disclosure Intention in Mobile Apps: The Mediating and Moderating Roles of Privacy Calculus and Temporal Distance

**DOI:** 10.3390/bs15030324

**Published:** 2025-03-06

**Authors:** Ming Chen, Meimei Chen

**Affiliations:** Glorious Sun School of Business and Management, Donghua University, Shanghai 200051, China; 1229100@mail.dhu.edu.cn

**Keywords:** perceived relevance, privacy reward mechanisms, privacy calculus theory, temporal distance, moderated mediation model

## Abstract

In digital societies, users’ privacy decisions not only affect personal information security and application sustainability, but also profoundly influence the formulation and enforcement of relevant laws. However, existing research mainly adopts a dichotomous perspective of rationality and bounded rationality, lacking an integrated framework to explain the complexity of users’ privacy decisions. Therefore, this study integrates privacy calculus theory and Psychological Distance Theory within the Elaboration Likelihood Model (ELM) framework to explore their mediating and moderating roles in the relationship between perceived privacy relevance and disclosure intention. To achieve the research objectives, we employed the vignette method to design an online survey with a 2 (perceived relevance: low vs. high) × 2 (temporal distance: near vs. distant) between-subjects design, ultimately collecting 293 valid responses. The conclusions are as follows: (1) perceived relevance positively affects users’ privacy disclosure intention, and perceived benefits and perceived risks play a partial mediating role between them; (2) contrary to individuals’ common preference for near temporal distance (immediate rewards), distant temporal distance (delayed rewards) have a more pronounced enhancing effect on the positive relationship between perceived relevance and perceived benefits; (3) the results excluded the influence of control variables such as gender, age, and education level on privacy disclosure intention. This study not only proposes an integrated analytical framework, revealing that users’ privacy decisions are jointly influenced by both rational and irrational factors, but also provides practical insights for application developers and regulatory agencies. Finally, we also discuss the limitations of this study and directions for future research.

## 1. Introduction

“Do you consent to granting TikTok to access the list of applications installed on your device?” Despite the fact that this request appears unrelated to its core functionality. We note that, in daily life, users frequently encounter app requests for privacy permissions. For instance, only after agreeing to the location privacy request can users enjoy more accurate location-based services (Momen et al., 2019). App privacy permissions constitute an access control mechanism for regulating application access to such system resources. As apps accumulate more data, privacy risks increase, leading to greater user concern for personal privacy security (Bongard-Blanchy et al., 2022). In response, regulations such as the “Regulations on Necessary Personal Information for Common Types of Mobile Internet Applications (2021)” have been issued in China to prevent apps from restricting access to basic functions when users refuse to provide non-essential information. Despite this, unauthorized collection of user information by apps continues to occur.

It is important to note that apps collecting user privacy information with low relevance to their core functions does not equate to collecting non-essential information as defined by law. For instance, device attributes, which may appear irrelevant to the core functions of most apps, are essential for the implementation of security features. This subtle difference can lead both app providers and users into a dilemma over privacy decisions. For the former, they need to reduce users’ privacy concerns while collecting information with low relevance. For the latter, on one hand, collecting low-relevance private information increases uncertainty about the necessity of authorization, which may exacerbate negative attitudes toward privacy decisions (Liu et al., 2022). On the other hand, despite these concerns, users continue to download and use apps in exchange for enjoyment (Shaw & Sergueeva, 2019), personalized services (Morosan & DeFranco, 2015), or societal benefits (Hassandoust et al., 2021).

Perceived relevance captures individuals’ perceptions of whether an information request is related to the transaction’s purpose (Culnan & Armstrong, 1999), which essentially reflects users’ pursuits of fairness. Additionally, Knijnenburg and Bulgurcu (2023) suggests that individuals use relevance to estimate the benefits of disclosure. This aligns with the assumption in many privacy studies that users are rational decision-makers. However, such rational judgments regarding privacy decisions appear to lack practical validation. Correspondingly, with the deepening exploration of the “privacy paradox”, researchers have found that individuals often face challenges in fully evaluating all risks and costs due to limited information, contextual constraints, or differences in cognitive abilities. Theories from other areas, such as the framing effect theory (Bongard-Blanchy et al., 2023) and the Elaboration Likelihood Model (Bansal et al., 2015), have been incorporated into privacy research. Given increasingly complex online environments and interface designs (Bahreini et al., 2022), integrating rational and bounded rationality perspectives into a unified framework to explain user privacy decision-making requires further exploration and development.

Additionally, monetary incentives significantly influence user decision-making. For instance, they can increase users’ willingness to provide personal information (Hui et al., 2007; L. Wang et al., 2017), or alleviate privacy concerns related to disclosing personal information in online transactions (Posey et al., 2010). However, while these studies have largely demonstrated the effect of monetary incentives on privacy decisions, the specific mechanisms and directions of this influence still require further investigation. To address the gaps identified in prior research, this study builds on the Elaboration Likelihood Model (ELM) and integrates privacy calculus theory (PCT) with Psychological Distance Theory (PDT) to develop a comprehensive framework that offers a unified explanation of privacy decision-making.

Based on the proposed framework, we found a positive relationship between perceived relevance and the intention to disclose, with perceived benefits and perceived risks partially mediating this relationship, consistent with the privacy calculus theory’s individual rational perspective. Additionally, by employing immediate and delayed rewards as proxies for different temporal distance contexts, our findings reveal that reward timing moderates the relationship between perceived relevance and perceived benefits. Notably, distant temporal distance (i.e., delayed rewards) significantly strengthens this relationship. The findings of our study offer valuable insights for various stakeholders in addressing privacy design challenges, providing guidance for improving privacy practices. In terms of theoretical contributions, our research proposes a framework that integrates two seemingly contradictory perspectives to explain the privacy decision-making process, suggesting that users’ privacy decisions may be shaped by the combined influence of both rational and irrational factors.

In the following section, we construct our research framework based on relevant theories. Section 3 provides a detailed description of the experimental process and sample characteristics. Section 4 presents the results of the statistical analysis. Finally, we summarize the conclusions of the study and discuss its limitations as well as future research directions.

## 2. Literature Review and Research Hypotheses

### 2.1. Privacy Calculus Theory (PCT)

The privacy calculus theory (PCT), introduced by Culnan and Armstrong (1999), provides a foundational framework for understanding privacy-related decision-making. It posits that individuals weigh perceived risks against perceived benefits when disclosing personal information, a concept rooted in social exchange theory (Donaldson & Dunfee, 1994) and utility maximization theory (Awad & Krishnan, 2006; Rust et al., 2002). Perceived risks involve potential losses from disclosing personal information, such as financial loss or privacy breaches, while perceived benefits include advantages like personalized services (Xu et al., 2011) or material incentives (Krafft et al., 2017).

Extensive empirical research has consistently validated the core principles of PCT. For instance, Maseeh et al. (2021) conducted a meta-analysis confirming that perceived risks positively correlate with privacy concerns, whereas perceived benefits exhibit a negative correlation. This relationship has been further corroborated by other studies (Cheng et al., 2021; Jabbar et al., 2023). However, the theory has faced criticism, particularly regarding the assumption of rational decision-making. Simon (1955) argued that human rationality is at best a rough approximation of complete rationality, a view often cited to critique PCT. Additionally, many researchers have questioned the role of “perceived risk”, suggesting that users do not necessarily engage in a risk calculation when disclosing privacy (Barth & De Jong, 2017). Instead, perceived benefits tend to have a greater impact on user satisfaction (Najjar et al., 2021).

Despite ongoing debates, PCT effectively encapsulates the fundamental principle of human decision-making: balancing potential benefits against perceived risks. Dienlin (2023) addressed criticisms by integrating general psychological models and a philosophy of science framework. This study posits that users evaluate privacy risks and benefits based on the relevance of requested information to the app’s core functionalities, leading to varied privacy behavior intentions. This aligns with PCT’s foundational principle of risk–benefit trade-offs. Therefore, PCT serves as a foundational framework for this research.

### 2.2. Psychological Distance Theory (PDT)

Psychological distance refers to the perceived likelihood of events or actions related to oneself across time, space, social connections, and hypotheticality. According to Construal Level Theory (CLT), psychological distance systematically influences how individuals construe objects or events, thereby shaping their behavior (Trope & Liberman, 2010). For example, H. Chen and He (2014) conducted three experiments and found that as psychological distance increased, participants were more inclined to choose delayed options in intertemporal decisions and preferred riskier options in risk-based choices. This implies that variations in psychological distance affect decision-making.

In the context of privacy decision-making, psychological distance plays a critical role. Bandara et al. (2021) analyzed the relationship between psychological distance, privacy concerns, and user information disclosure through subjective surveys, confirming the direct impact of psychological distance on user information disclosure. Temporal distance, a classic dimension of psychological distance, refers to an individual’s perception of the proximity of an event or object to the present moment (Liberman & Trope, 1998; Trope & Liberman, 2003). Acquisti and Grossklags (2003) applied the concept of temporal discounting to explain the privacy paradox, arguing that perceived temporal distance diminishes the weight individuals place on the long-term benefits of privacy protection when making disclosure decisions.

Reward timing, as a manifestation of temporal distance, significantly influences privacy disclosure intentions and decision-making processes. Specifically, when rewards are immediate, the perceived temporal distance is short, leading to more concrete, immediacy-focused decision-making. In contrast, when rewards are delayed, the perceived temporal distance increases, prompting individuals to adopt a more abstract, long-term oriented approach to decision-making (Trope & Liberman, 2010). This shift in decision-making style highlights how temporal distance influences the way individuals assess the significance of their choices. Hallam and Zanella (2017) found that individuals without prior experiences of privacy invasion are more likely to sacrifice privacy for immediate gratification. Building on these insights, this study also seeks to explore the impact of temporal distance on users’ privacy risk–benefit trade-offs, aiming to uncover the role of psychological distance in privacy decision-making.

### 2.3. Elaboration Likelihood Model (ELM)

Permission requests aim to persuade users to share personal information through a complex user information-processing mechanism. Levin et al. (1998) introduced the Elaboration Likelihood Model (ELM), which provides a cognitive framework for understanding this process. The ELM differentiates between two distinct information-processing pathways: the central route and the peripheral route. The central route involves logical and rational analysis, consistent with the privacy calculus perspective, while the peripheral route relies on heuristic evaluations, such as emotions, contextual factors, or social cues (Zhou, 2017).

In the context of privacy disclosure, users’ privacy concerns regarding data collection and access are often regarded as central cues in the privacy calculus process (Bansal et al., 2015). Yu et al. (2020) employed the ELM to analyze how perceived privacy risks and concerns influence disclosure intentions, treating perceived risk as a central cue. Similarly, permission relevance, which shapes assessments of benefits and risks, is treated as a central cue in this study. However, decision-making in privacy disclosure is not purely rational; users often rely on heuristic processing, particularly when evaluating reward timing. Immediate gratification can lead to self-control issues (O’Donoghue & Rabin, 2000), where immediate rewards overshadow potential privacy risks (Bandara et al., 2020). Notably, when assessing reward timing, individuals often use heuristics, prioritizing readily available information over systematic analysis (Marzilli Ericson et al., 2015), aligning with ELM’s definition of peripheral cues. Therefore, we treat the temporal distance of privacy disclosure rewards as a peripheral cue that influences users’ privacy decisions.

In summary, ELM’s strength lies in its ability to integrate both rational (central route) and heuristic (peripheral route) decision-making processes, capturing the complex interplay of rationality and behavioral biases in privacy decisions. Furthermore, ELM provides clear guidance for variable operationalization (such as central vs. peripheral cues), making it a robust theoretical framework for this study.

### 2.4. Research Hypothesis

#### 2.4.1. Perceived Relevance and Privacy Disclosure Intention

Previous research on privacy sensitivity suggests that requesting sensitive information may elevate users’ privacy concerns, thereby diminishing their willingness to share such information (Malhotra et al., 2004). Building on this notion, Li et al. (2010) argue that users are more likely to perceive a website that collects only information relevant to its core business as more likely to respect and protect their privacy. In contrast, websites that request unrelated information may raise concerns about potential misuse. They further define perceived relevance as “the degree of relevance between the permissions requested by an app and its core functionalities”.

The shift from sensitivity to relevance indicates that identical privacy disclosure requests may be perceived as either more acceptable or less acceptable depending on the context (Malheiros et al., 2013). For instance, users of banking applications may consider providing personal identification information as reasonable, whereas in contexts such as music player apps, such requests may be seen as high-risk behavior. The relevance of permission requests highlights the role of specific privacy contexts over the sensitivity of the information itself. Zimmer et al. (2010) argued that relevance is context specific, affects perceptions of utility, and varies from one individual to another. Perceived relevance thus plays a crucial role in shaping users’ privacy disclosure. Leom et al. (2021) confirmed that perceived relevance is significantly positively correlated with self-disclosure. Therefore, we propose the following hypothesis:

**Hypothesis** **1** **(H1).** *Perceived relevance positively influences users’ intention to disclose privacy*.

#### 2.4.2. The Mediating Role of Perceived Risk and Perceived Benefits

Perceived relevance is likely to be positively associated with an individual’s intention to disclose privacy. However, the relationship between them could be mediated by other factors. Users may have concerns regarding the collection process, secondary usage, and accessing of personal information (Smith et al., 1996). These concerns contribute to the perceived potential losses individuals perceive when disclosing information to relevant entities, defined as perceived privacy risks (Malhotra et al., 2004). Harborth and Pape (2020) found that higher privacy concerns lead to a perception of privacy risks when studying users’ willingness to use privacy-enhancing technologies. In addition, the perceived severity of the negative consequences of security issues also exacerbates users’ privacy concerns, while the enhancement of trust (van der Schyff & Flowerday, 2023) and perceived control (Harborth & Pape, 2020) weakens these concerns.

As previously discussed, the relevance between permission types and the core functionalities of an app aligns more closely with the reality of privacy requests. By applying for permissions related to the app’s operation and core functions (e.g., a food delivery app applying for geolocation information), apps can reduce the perceived risks associated with disclosure. In contrast, app providers that collect information that is not relevant to their business send higher risk signals to users (Zimmer et al., 2010).

Perceived risk, a core factor in privacy calculus theory, reflects users’ cost considerations when treating privacy as a commodity during exchange processes. In order to mitigate the risky consequences of privacy disclosure, users seek privacy protection. R. Chen (2013) conducted an empirical study through questionnaire surveys to show that the greater the user’s perceived privacy risk, the stronger the attitude towards privacy protection. Conversely, privacy risks can inhibit users’ willingness to disclose their privacy. In addition, Hajli and Lin (2016) investigated the privacy attitudes of US users towards three social apps, and found that privacy risks negatively affect users’ willingness to disclose information, and similar conclusions are also supported by privacy research in the context of IT-enabled ride-sharing (Cheng et al., 2021). In light of our previous discussion, we propose the following hypothesis:

**Hypothesis** **2** **(H2).** *Perceived risks will mediate the relationship between perceived relevance and privacy disclosure intention. Specifically, perceived relevance negatively affects perceived risk (H2a), while perceived risk reduces users’ intention to disclose privacy (H2b)*.

Perceived benefit, as another core factor in PCT, reflects users’ subjective perception of the benefits they can obtain from disclosing their information. Users’ perceptions of benefits are not limited to monetary incentives (Xie et al., 2006), but also include personalized product customization or recommendations (Smith et al., 2011). Cheung et al. (2015) subdivided perceived benefits into four aspects when studying self-disclosure on social networking sites (SNS): convenience of maintaining existing relationships, new relationship building, self-presentation, and enjoyment. In summary, the functions and services provided by the app, along with the economic and emotional value they bring to users, collectively constitute users’ perceived benefits. In the field of advertising and marketing, personalized advertising can reduce information overload and save consumers’ time and energy, which is seen as a benefit and helps to alleviate their privacy concerns (Zhu & Chang, 2016). Therefore, reasonably requesting permissions relevant to the core functionalities of the app will enhance users’ trust and consequently strengthen their perception of benefits. Moreover, higher perceived relevance implies that disclosing such information allows users to maximize the use of the app’s functionalities, which they consider as important privacy benefits.

In contrast to perceived risk, perceived benefits show a positive relationship with privacy disclosure, which aligns with the fundamental logic between users’ online behavior and their perception of benefits. For example, if shoppers are offered online discounts or promotions, they are more likely to disclose their personal information (Dinev & Hart, 2006; Hui et al., 2007). Sun et al. (2019) found that for users of social e-commerce, perceived benefits had a positive impact on information disclosure behavior. In conclusion, the higher the perceived benefits for users, the stronger their willingness to engage in privacy disclosure to obtain greater benefits, ultimately translating into actual behavior. In light of our previous discussion, we propose the following hypothesis:

**Hypothesis** **3** **(H3).** *Perceived benefits will mediate the relationship between perceived relevance and privacy disclosure intention. Specifically, perceived relevance positively affects perceived benefits (H3a), while perceived benefits increase users’ intention to disclose privacy (H3b)*.

#### 2.4.3. The Moderating Effect of Temporal Distance

Individuals’ perceptions of the benefits of privacy disclosure primarily depend on their speculations or expectations regarding future outcomes. Perceived relevance allows individuals to more concretely evaluate the potential benefits of privacy disclosure. In this study, we propose that temporal distance negatively moderates this positive relationship. In practice, individuals often demonstrate impatience in their time preferences, tending to choose immediate smaller rewards over delayed larger ones (Burghoorn et al., 2024). This preference has important implications for marketing, where product benefits are often framed in either near-term or distant future perspectives, under the assumption that consumers evaluate these benefits differently based on temporal proximity (Youn & Kim, 2018).

In the context of privacy, research on immediate gratification also shows that individuals tend to prioritize immediate benefits while overlooking future privacy risks, leading to a reduction in privacy-protective behaviors (Acquisti, 2004). Hallam and Zanella (2017) also found that users are more inclined to obtain immediate and tangible social benefits compared to potential privacy violations that might occur in the distant future. Additionally, J. Zhang et al. (2024) indicate that immediate rewards are more likely to induce impulsive decision-making, which in turn increases privacy-disclosing behaviors. These findings suggest that in contexts characterized by high temporal distance (e.g., delayed rewards), users’ perception of benefits may weaken. Therefore, we propose the following hypothesis:

**Hypothesis** **4** **(H4).** *The mediating effect of perceived relevance on privacy disclosure intention through perceived benefits is negatively moderated by temporal distance*.

The influence of temporal distance on users’ risk perception has been extensively validated. Studies indicate that users tend to prefer rewards that occur sooner rather than later, with the allure of immediate rewards often outweighing their concerns about future privacy risks (Bandara et al., 2018, 2020). In other words, as temporal distance increases (e.g., with delayed rewards), concerns about risks may intensify. According to the implicit-risk hypothesis in the field of delay discounting, greater temporal distance to reward realization heightens uncertainty, which in turn elevates individuals’ perceived risk levels (Jiang & Dai, 2021; Johnson et al., 2020). This effect may also be pronounced in the context of privacy permission requests. Specifically, when users perceive that the permissions requested by an application are highly relevant to its core functionality, delayed rewards could potentially amplify their risk assessments, which are otherwise relatively low. Consequently, we propose the following hypothesis:

**Hypothesis** **5** **(H5).** *The mediating effect of perceived relevance on privacy disclosure intention through perceived risk is negatively moderated by temporal distance*.

**Figure 1:**
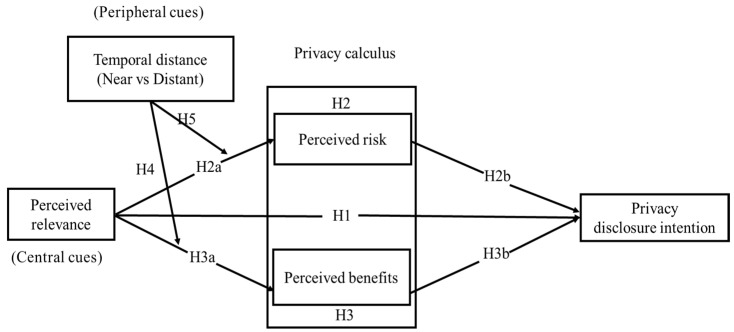
The research model.

Finally, we included gender, age, and education level as control variables. Based on the review of three theories and our proposed hypotheses, we developed the following research model (see Figure 1).

## 3. Method

To validate the proposed research model, this study employed the vignette method. This research approach provides respondents with brief, specific scenarios or situations related to the study before they respond, forming a systematic combination conducive to research. It combines the advantages of experimental research and traditional subjective surveys, achieving a balance between internal and external validity issues (Atzmüller & Steiner, 2010).

Given that this study employs the vignette method, conducting pre-surveys is essential before using image materials to ensure participants fully understand the purpose and content of the research. The pre-survey aims to address the following three issues: Firstly, it determines whether participants can clearly perceive how the timing of privacy disclosure rewards is expressed. Secondly, it verifies the specific types of rewards provided to users upon disclosing privacy and establishes the value of these rewards over varying temporal distances. Lastly, it clarifies the specific situations of two types of privacy information requests, namely whether personal privacy is highly or scarcely relevant to the application’s core functions or services. To achieve the aforementioned research objectives, we conducted the five pre-surveys described in Section 3.1 from mid-January to the end of March 2024.

### 3.1. Experimental Stimulus

In the research of bundled sales, Karataş and Gürhan-Canli (2020) employed “immediate” and “one month later” as distinct representations of temporal distance. Similarly, Kostelic (2021) employed these terms to influence users’ purchase decisions across various timeframes. Combining these studies, we conducted an online survey (pre-survey (1)) and found that compared to offering privacy disclosure rewards “one month later”, 98.6% of respondents (*n* = 69) perceived the psychological distance of offering such rewards “immediately” as closer. Therefore, we adopt “immediate” and “one month later” as expressions indicating different temporal distances for the redemption of privacy disclosure rewards.

Currently, app incentives for users to disclose personal information primarily involve providing discounts, coupons, and benefits. In our pre-survey (2), we initially asked participants to select their preferred form of privacy disclosure reward across different types of apps (such as online payment, online shopping, online food delivery, and online travel). Subsequently, participants were asked whether they believed their chosen reward would influence their own information disclosure behavior. Respondents have the highest preference for cash rewards, and more than 80% of users who choose cash rewards believe that this type of incentive would impact their privacy decisions (*n* = 96). We believe that users will only care about the timing and value of reward redemption if they are interested in the reward content. Hence, we have chosen cash (red envelope) rewards as the incentive for privacy disclosure.

The concept of temporal discounting reveals how people assess the value of future rewards that change over time. To determine the values of rewards redeemed at different times, we conducted pre-survey (3) (*n* = 76) and found that if an app delays the delivery of a 100-point reward by one month due to system malfunction, 44.1% of participants believe that the compensation should be increased to 200 points, while 28.5% chose 300 points, indicating that the compensation one month later should be twice that of the immediate reward. Further, when we replaced the point reward with a red cash envelope in pre-survey (4) (*n* = 80), 45.6% agreed that a one-month delay merits doubling the reward to CNY 10 from the initial CNY 5. Based on this, we set the reward standards as follows: CNY 5 for immediate redemption and CNY 10 after one month.

In pre-survey (5), focusing on the online shopping app, we defined two scenarios for privacy permission requests (*n* = 105). The findings highlighted that geolocation permissions exhibited the highest relevance to shopping apps (M = 5.363), whereas app list permissions showed lower relevance (M = 3.838), with a significant difference between them (t = 6.093, *p* < 0.001). Based on these five pre-surveys, we ultimately determined four illustrative materials: “The online shopping app requests access to your Geolocation (or List of applications) and will immediately provide you with a 5 CNY cash red envelope”; “The online shopping app requests access to your Geolocation (or List of applications) and will provide you with a 10 CNY cash red envelope one month later”.

### 3.2. Experimental Procedures

Before collecting data, we used G-Power 3.1 to calculate the minimum sample size required. Drawing on prior research, we set α = 0.05 (two-tailed), 1 − β = 0.80, and the minimum sample size was found to be 155. To ensure data reliability, we aimed to collect more than this minimum.

During the data collection phase (from 2 July 2024 to 17 July 2024), we designed a between-subject experiment with a 2 (perceived relevance: low vs. high) × 2 (temporal distance of reward redemption: immediate vs. one month later) design. The survey consisted of three sections: the first section introduced the experimental scenario, where participants were informed that an online shopping app offers cash rewards for agreeing to different permissions. These rewards would be automatically credited to the participant’s shopping account and could be withdrawn later. The scenario was framed to simulate realistic app behaviors in terms of permission requests, aiming to enhance participants’ perceptions of the authenticity of the reward for granting authorization.

In the second parts of this survey, each respondent was asked to answer questions related to privacy disclosure tendency (Li et al., 2010; Liu et al., 2022), perceived risk (Zhou, 2011), perceived benefits (Sun et al., 2014; T. Wang et al., 2016) and the perceived relevance (Hajli & Lin, 2016). A 7-point Likert scale was used to measure the four variables in the model (see Appendix A Table A1). After completing the assessment of the measured variables, we asked participants to rate the perceived temporal distance of two reward distributions, with 1 indicating “very near” and 5 indicating “very distant”. In the third section of the questionnaire, we also gathered demographic details, including respondents’ gender, age and educational level (refer to Table 1). To reach a broader sample, the survey was conducted online and disseminated through social platforms like WeChat. Additionally, participants were offered cash incentives between 1 and 2 CNY to boost their motivation and ensure reliable responses to the survey questions.

### 3.3. Data Screening

#### 3.3.1. Descriptive Analysis

Table 1 reveals a notable gender disparity in respondent sample, with more females (155) than males (138). Acknowledging this, we included gender as a key control variable in subsequent analyses. The model results indicated that gender did not significantly impact the dependent variable, confirming effective control for gender differences. Furthermore, the proportion of respondents aged 19–35 reached 73.7%, while those aged 36 and above accounted for 24.5%. Lastly, 230 respondents held undergraduate degrees, indicating a generally high level of education within the surveyed population.

#### 3.3.2. Common Method Bias and Non-Response Bias

When gathering responses from the surveyed population, variations in item characteristics, questionnaire content, and the questionnaire environment can all contribute to a degree of response bias (Reio Jr., 2010). As a control for common method bias, we employed the unmeasured latent marker construct (ULMC) method for validation that proposed by Chin et al. (2012). This method extracts a truer common variance compared to the basic common latent factor method, as it identifies the common variance between unrelated latent factors. The data results from Table 2 suggest that incorporating the common latent factor in Model 2 did not substantially improve the model fit indicators compared to Model 1. This implies that the sample data in this study did not demonstrate significant issues related to common method bias (Richardson et al., 2009). Additionally, we extracted the first 15% and the last 15% of data samples for the four categorical scenarios and conducted an independent samples *t*-test on the means of the four study variables. The statistical analysis revealed no significant difference between the two, indicating that response bias was not a concern in this study. Finally, variance inflation factor (VIF) values were used to assess collinearity in this model. The VIF scores for the model’s endogenous latent variables ranged from 2.118 to 3.031, all below the threshold of 5 recommended by Hair et al. (2021), confirming the absence of collinearity issues.

#### 3.3.3. Measurement Model

Table 3 presents the results of the measurement model. First, the Cronbach’s α coefficients and composite reliability (CR) values for all constructs exceed the standard threshold of 0.70, indicating high internal consistency. Additionally, most factor loadings surpass the recommended value of 0.70. Combined with the average variance extracted (AVE) estimates, which range from 0.711 to 0.893, these results confirm the convergent validity of the constructs.

Furthermore, discriminant validity was assessed using the Heterotrait–Monotrait ratio (HTMT) proposed by Henseler et al. (2015) and the Fornell–Larcker criterion (Fornell & Larcker, 1981). The HTMT criterion requires that correlations between constructs remain below 0.85. As shown in Table 4, all values meet this criterion, indicating satisfactory discriminant validity. Moreover, the square roots of the AVE values, presented along the diagonal in Table 5, exceed the correlations between constructs, further supporting the discriminant validity of the measurement model.

## 4. Results

### 4.1. Manipulation Check

We employed an independent sample *t*-test to determine if there was a difference in the relevance of geographic location and app listings for online shopping apps. The data results indicate that geolocation (Mean = 4.88; SD = 1.59) is considered more advantageous for facilitating the functionality of online shopping apps compared to application listings (Mean = 4.01; SD = 1.57). A significant difference in perceived relevance exists between these two types of privacy and shopping apps (t = −4.686, *p* < 0.001). Additionally, we performed independent sample *t*-tests for the perceived relevance of privacy permissions at different time intervals—“immediately” and “one month later”—for both geolocation and application listings. As shown in Table 6, respondents’ views on the relationship between privacy types and app relevance remain unchanged regardless of the timing of reward redemption; all *p*-values are greater than 0.05. Finally, we observed that participants perceived a near temporal distance in the context of immediate rewards (Mean = 2.30) compared to a distant temporal distance in the context of delayed rewards (Mean = 3.94), with a significant difference between them (t = −4.440, *p* < 0.001). Therefore, the operation carried out using the vignette method is supported by data.

### 4.2. Preliminary Analyses

We present the mean, standard deviation, and correlation coefficients for each research variable in Table 7. The findings reveal significant associations among all variables. Notably, perceived relevance strongly predicts privacy disclosure intention (a = 0.754). As anticipated, perceived risk demonstrates a significant negative association with both perceived relevance and privacy disclosure intention. Conversely, perceived benefits show a significant positive association with both. Recognizing the Pearson correlation coefficient’s limitation in capturing only linear relationships and its incapacity to infer causality, we proceed with further hypothesis testing to validate the proposed model.

### 4.3. Hypothesis Testing

#### 4.3.1. Direct and Indirect Effects

To investigate both the direct influence of perceived relevance (PRE) on privacy disclosure intention (PDI) and the indirect effects mediated by two variables in the privacy calculus process: perceived risk (PR) and perceived benefit (PB), we employed the PROCESS macro (Model 4) to conduct a simple mediation model analysis. After controlling for variables such as gender, age, and education level, Model 1 in Table 8 (with PR as the dependent variable) shows that perceived relevance (PRE) has a significant negative impact on perceived risk (PR) (b = −0.615, SE = 0.041, *p* < 0.001), thus supporting H2(a). In addition, in Model 3 (PDI as the dependent variable), it was further found that there was a significant negative correlation between PR and PDI (b = −0.513, SE = 0.051, *p* < 0.001), and H2(b) was also verified. Similarly, there is a significant positive correlation between PRE and PB in Model 2 (PB as the dependent variable), which in turn significantly positively affects PDI, thus providing data support for H3(a) and H3(b). After incorporating the two mediating variables, the positive relationship between PRE and PD remains significant (b = 0.298, SE = 0.051, *p* < 0.001). This implies that PR and PB partially mediate the relationship between PRE and PD, thus validating H1, H2, and H3.

Further bootstrapping results show that the total effect value of PRE on PDI is 0.826 (95% CI [0.743, 0.909]), with a direct effect of 0.298 (95% CI [0.197, 0.399]). The mediated effect value with PR as the mediator is 0.316 (95% CI [0.213, 0.422]), and the mediated effect value with PB as the mediator is 0.212 (95% CI [0.129, 0.306]). None of these intervals include 0, which means that the mediated effects account for a total of 63.9% of the explained variance.

#### 4.3.2. Testing of the Moderated Mediation Model

We utilized the PROCESS macro (Model 7) with 5000 bootstrap samples, following the methodology outlined by Hayes (2017), to test the moderated mediation model. In the statistical analysis, the “list of applications” was coded as “0” to represent the low-relevance condition, while “location” was coded as “1” to represent the high-relevance condition. A similar coding scheme was applied to the temporal distance of reward redemption.

The statistical results in Table 9 show that temporal distance does not moderate the negative relationship between perceived relevance and perceived risk (b = 0.051, SE = 0.083, t = 0.614, 95% CI [−0.092, 0.216]). Additionally, the index of moderated mediation (b = −0.026, SE = 0.043, 95% CI [−0.116, 0.056]) includes 0, suggesting that H5 is not supported by the data. Conversely, the analysis reveals a significant positive moderating effect of temporal distance on the relationship between perceived relevance and perceived benefits (b = 0.136, SE = 0.062, t = 2.184, *p* < 0.05, 95% CI [0.013, 0.258]). This finding contradicts our initial hypothesis, leading to the rejection of H4. Secondary analyses using PROCESS macro (Model 8) further confirm these findings and show that temporal distance does not moderate the positive relationship between perceived relevance and disclosure intention (b = 0.018, SE = 0.067, t = 0.276, 95% CI [−0.113, 0.149]), providing insights beyond our initial hypotheses.

**Figure 2:**
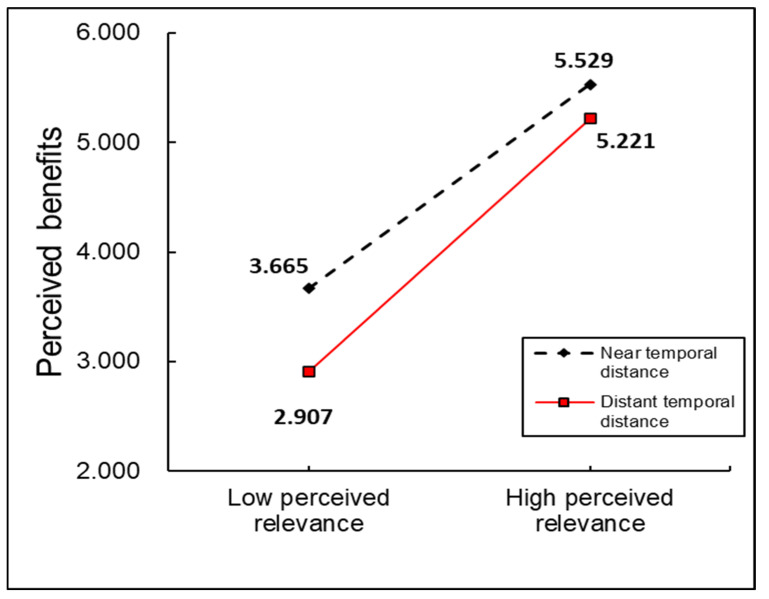
Temporal distance as a moderator of the relationship between perceived relevance and perceived benefits.

To further investigate potential moderated mediation effects related to H4, we conducted additional analyses. As shown in Table 10, temporal distance significantly moderates perceived benefits under both immediate and delayed reward conditions. Specifically, under the condition of immediate reward redemption, b = 0.183, SE = 0.039, with a 95% CI [0.110, 0.265], whereas under reward redemption one month later, b = 0.227, and SE = 0.048, with a 95% CI [0.138, 0.324]. A simple slope analysis (see Figure 2) confirms that under delayed reward conditions, the relationship between perceived relevance and perceived benefits is significantly stronger (b = 0.703, SE = 0.042, 95% CI [0.621, 0.786]) compared to immediate reward conditions (b = 0.568, SE = 0.046, 95% CI [0.477, 0.658]).

Finally, the index of moderated mediation was also found to be significant (b = 0.044, SE = 0.023, 95% CI [0.002, 0.091]). Additionally, pairwise contrasts between conditional indirect effects reveal that the indirect effect under the delayed condition is significantly higher than under the immediate condition (contrast = 0.0440, 95% CI = [0.0008, 0.0953]), further supporting the moderating role of temporal distance.

## 5. Discussions and Implications

### 5.1. Summary of Key Findings

This study validates a significant positive relationship between perceived relevance and privacy disclosure intention (b = 0.296, *p* < 0.001), aligning with the findings of Knijnenburg and Bulgurcu (2023). Zimmer et al. (2010) conceptualized relevance as a contextual privacy factor that influences users’ perceptions of information utility. Specifically, high relevance not only enhances users’ perceptions of the fairness of information requests (Li et al., 2010), but also reduces perceived uncertainty among users (Liu et al., 2022). Conversely, applications requesting low-relevance personal information may heighten perceived privacy risks, as such requests surpass users’ expected privacy boundaries, thereby increasing the likelihood of unauthorized misuse. Furthermore, according to the Elaboration Likelihood Model (ELM), which posits that decisions based on central cues are highly stable and resistant to reversal, this explains why temporal distance did not significantly moderate this relationship in our supplementary analysis.

Privacy calculus serves as a partial mediator, demonstrating how permission request relevance fundamentally shapes users’ privacy-related decision-making processes. When apps request permissions with low relevance, users’ perceptions of privacy leakage risk significantly increase. This finding aligns with Li et al. (2010), who identified a negative correlation between perceived relevance and privacy risk beliefs. Furthermore, low relevance in data requests is often associated with heightened feelings of privacy invasion (Zhu & Chang, 2016). Our analysis reveals a notably stronger indirect effect of perceived risk (b = 0.315) compared to perceived benefits (b = 0.212), indicating that users prioritize “risk avoidance” over “benefit pursuit” in their privacy disclosure decisions based on perceived relevance.

It is worth noting that, although perceived relevance negatively influences perceived risk, temporal distance did not significantly moderate the relationship between them in this study. We offer the following explanations: A frame is viewed as a mental model used by individuals to address decision-making problems. It encompasses not only the details intrinsic to the decision-making problem but also includes relevant contextual information (Johnson-Laird, 1983). Different descriptions of the same objective problem can lead individuals to develop distinct behavioral preferences and make different choices (Kahneman & Tversky, 1979). In this study, the information frame set by the researchers emphasized the rewards of privacy disclosure, which may have contributed to the lack of moderation by temporal distance on the relationship between perceived relevance and perceived risk. Furthermore, in contexts where perceived relevance serves as the central cue, perceived risk may exhibit stability and be less susceptible to the influence of temporal distance. This could be another reason for the non-significant moderating effect.

Conversely, temporal distance moderated the relationship between perceived relevance and perceived benefits. Specifically, the positive association between perceived relevance and perceived benefits was strengthened when the redemption time for cash rewards was distant. Temporal discounting theory suggests that individuals may prefer delayed benefits because the perceived value of the outcome increases over time (Loewenstein, 1987). In today’s app-driven information society, disclosing privacy information highly relevant to app functionalities has increasingly become the only viable option. In this context, users may choose to delay reward redemption to exchange for greater value. Furthermore, Construal Level Theory (CLT) provides additional cognitive explanations for this result. According to CLT, immediate rewards trigger concrete thinking (Bandara et al., 2018), while delayed rewards promote more abstract cognitive processing (Wakslak et al., 2006). High-level construal of a given behavior are prioritized in long-term decision-making, whereas low-level construal is emphasized in short-term decision-making (Eyal et al., 2009; Trope & Liberman, 2000). Under these circumstances, users tend to view the relationship between perceived relevance and perceived benefits as a form of fair exchange. Delayed rewards extend the temporal context of privacy disclosure, enabling users to attribute deeper meaning to their actions (e.g., altruistic behavior, value identification).

Finally, our results show that all control variables have no significant impact on the intention to disclose, yet this does not mean that the researchers should ignore the influence of individual differences on privacy decisions. M. Zhang et al. (2020) found that women often have higher privacy concerns regarding the use of location-based services (LBS). Ioannou et al. (2021) also found that, compared to younger users, older individuals often lack the knowledge and skills to utilize information services and products, which may lead to lower levels of privacy concern. Therefore, future research could explore how different privacy contexts interact with individual characteristics to affect privacy decisions.

### 5.2. Theoretical Implications

Firstly, this study introduces an integrative perspective on the privacy decision-making process, highlighting the joint role of both rational and irrational factors as the core mechanism driving privacy-related decisions. This perspective contrasts with most prior studies, which often treated these two approaches as mutually exclusive. Our proposed theoretical framework not only reaffirms the robust explanatory power of privacy calculus theory, but also supports the legitimacy of the principle of minimization in privacy laws governing app permission requests across various jurisdictions. Specifically, within the context of this research, users tend to evaluate privacy choices by balancing risks and benefits based on the relevance of requested permissions. If apps frequently request permissions that are less relevant to their core functions, this could heighten users’ perceived privacy risks, potentially resulting in unpredictable long-term challenges for the sustainable development of such apps.

Secondly, Solove (2021) argued that drawing broad conclusions about the “privacy paradox” based on individuals’ decisions regarding personal privacy risks in specific contexts involves a logical flaw. Our research framework serves as a complementary response to this perspective. On one hand, our study shows that perceived risk plays a more significant mediating role than perceived benefits in users’ privacy disclosure decisions under perceived relevance, which challenges some scholars’ claims that users do not engage in risk trade-offs to support the privacy paradox; on the other hand, our findings reveal that temporal distance positively moderates the relationship between perceived relevance and benefits, indicating that peripheral cues can enhance or even reverse rational decision-making effects in certain contexts. Importantly, this study does not exaggerate the role of peripheral cues in privacy decision-making, but treats them as moderating variables. Both rational and bounded-rational factors are interconnected, collectively shaping the complexity of privacy decision-making.

Finally, we employed the vignette method, which is well-suited for investigating contextual problems. To ensure the scientific rigor of the experimental scenarios, we conducted a series of pre-surveys. These methodological and procedural efforts not only enhance the reliability of our findings but also provide meaningful references for future research in the field of privacy-related issues.

### 5.3. Practical Implications

Based on our research conclusions, we put forward the following practical suggestions:

For app developers, on the one hand, it is essential to clearly define core functions and the user privacy information requirements for the app’s operation. The negative relationship between perceived relevance and perceived risk constrains unlimited collection of user privacy. Therefore, developers should gather user feedback prior to launching the app. When certain information is required but users deem it irrelevant, designers can add incentives within the law to incentivize users to disclose privacy. This approach is particularly important in regions with strict privacy regulations, such as countries that implement the European Union’s General Data Protection Regulation (GDPR), and among user groups who are more sensitive to privacy.

On the other hand, developers can enhance user experience by continuously optimizing reward redemption time and value schemes. Liu et al. (2022) found that lower relevance increases users’ uncertainty judgments. Therefore, when the app requests information that is not highly relevant to the core business but is crucial for service optimization, emphasizing the immediacy of reward redemption can help alleviate user distrust. This strategy may be particularly effective in emerging markets, where users’ trust in digital platforms tends to be lower. Furthermore, recognizing that some users will abstract their privacy disclosure behaviors, the app may consider offering non-monetary additional incentives, such as customized identity labels, to enhance users’ perception of the value of their privacy actions, thus transcending a simple understanding of privacy–service exchange behavior.

In addition, although the Chinese government has established clear regulations through relevant laws regarding the basic information required by various apps to function effectively, the phenomenon of excessive authorization requests persists. Therefore, policymakers should take into account the diverse contexts of app operations, including variations in user demographics, regional privacy expectations, and the specific nature of app services, in order to develop comprehensive privacy regulation strategies. Finally, users should recognize their own needs and, taking factors such as the platform’s reputation into account, carefully evaluate the legitimacy of the app’s privacy permission requests to safeguard their privacy and security.

## 6. Research Limitations and Future Directions

Although this study has come to some interesting conclusions, there are still some research limitations and future directions to be explored: First of all, we employed cash rewards as an incentive for privacy disclosure in this study. However, this approach may have limitations in ecological validity, as users in real-world contexts rarely receive direct monetary compensation for providing personal information. Instead, they are more likely to disclose their information in exchange for non-monetary incentives, such as coupons, discounts, or membership benefits. Consequently, our experimental design may not fully reflect the privacy decision-making process in real-world scenarios. To address this limitation, future research could explore incentive mechanisms that more closely align with real-world applications. Furthermore, examining how users’ privacy decisions vary under different incentive structures could provide deeper insights into the complexity of privacy disclosure behavior.

Secondly, while the immediacy or delay of rewards is commonly used to manipulate temporal distance, the simultaneous involvement of both time and monetary factors may affect the interpretability of the research results. Therefore, we suggest that future studies design a singular temporal manipulation condition to more purely test the impact of temporal distance on privacy decisions.

Thirdly, we did not incorporate ELM’s inherent individual analysis capability as a moderating variable in model construction. We assumed uniform privacy literacy among users. Research by Rosenthal et al. (2020) has found that as privacy literacy increases, the negative impact of privacy concerns on the acceptance of personalized information decreases. In our study, we take education level as a control variable into consideration, while the education level is not synonymous with privacy literacy. Therefore, future research should consider how diverse user traits may affect privacy behaviors in various contexts. Furthermore, as this study is based on a small sample, future research could try to use larger sample sizes to enrich and validate the conclusions.

At last, although the vignette method has certain advantages, it cannot completely eliminate the influence of individual participants’ subjective biases on the results. Therefore, conclusions drawn from this research method in the future can be further verified by objective indicators. With the advancements in neuroscience, neuro-privacy has become a new trend in privacy research. Techniques such as event-related potential (ERP) or eye-tracking provide new ways to obtain objective physiological indicators.

## Figures and Tables

**Table 1:** Demographic characteristic of the participants (*n* = 293).

Gender		Age		Educational Level	
Category	Number	Category	Number	Category	Number
Male	138	<18	5	High school and below	9
Female	155	19–24	74	Undergraduate	230
		25–30	68	Master’s degree and above	54
		31–35	74		
		36–40	37		
		>40	35		

**Table 2:** Common method bias test.

	χ^2^/df	RMSEA	SRMR	GFI	AGFI	CFI	NFI
CFA model1 (Without CLF)	1.540	0.043	0.016	0.961	0.937	0.993	0.981
CFA model2 (CLF Added)	1.556	0.044	0.014	0.966	0.935	0.994	0.984
Δ (model1 − model2)	−0.016	−0.001	0.002	−0.005	0.002	−0.001	−0.003

Note: RMSEA: root mean square error of approximation; SRMR: standardized root mean square residual; GFI: goodness-of-fit index; AGFI: adjusted goodness-of-fit index; CFI: comparative fit index; NFI: normed fit index.

**Table 3:** A summary of the measurement model (*n* = 293).

	Mean	SD	Factor Loading	Cronbach α	CR	AVE
PDI1	4.181	0.043	0.952	0.961	0.962	0.893
PDI2	4.092	0.046	0.938			
PDI3	4.256	0.045	0.944			
PR1	4.317	0.046	0.911	0.943	0.943	0.807
PR2	4.614	0.058	0.895			
PR3	4.590	0.063	0.886			
PR4	4.464	0.057	0.900			
PB1	4.420	0.110	0.659	0.870	0.878	0.711
PB2	4.215	0.052	0.928			
PB3	4.334	0.056	0.915			
PRE1	4.546	0.089	0.835	0.902	0.909	0.833
PRE2	4.413	0.083	0.985			

Note: PDI: privacy disclosure intention; PR: perceived risk; PB: perceived benefits; PRE: perceived relevance.

**Table 4:** Heterotrait–Monotrait Ratio (HTMT).

	**PDI**	**PR**	**PB**	**PRE**
PDI	-			
PR	0.843	-		
PB	0.846	0.796	-	
PRE	0.800	0.709	0.844	-

Note: PDI: privacy disclosure intention; PR: perceived risk; PB: perceived benefits; PRE: perceived relevance.

**Table 5:** Fornell and Larcker discriminant validity.

	PDI	PR	PB	PRE
PDI	0.945			
PR	−0.801	0.898		
PB	0.779	−0.721	0.843	
PRE	0.754	−0.655	0.766	0.913

Note: The diagonal value represents the square root of the AVE.

**Table 6:** Perceived privacy relevance for online shopping apps with different reward timings.

	Reward Timing	Mean	SD	*t*-Value	*p*
Geolocation	Immediate	5.02	1.51	1.119	0.265
	One month later	4.74	1.66		
List of apps	Immediate	4.22	1.54	1.496	0.137
	One month later	3.81	1.59		

**Table 7:** Means, standard deviations, and correlation matrix of all variables.

	Mean	SD	PDI	PR	PB	PRE
PDI	4.191	1.791	1			
PR	4.488	1.531	−0.801 **	1		
PB	4.316	1.410	0.779 **	−0.721 **	1	
PRE	4.483	1.639	0.754 **	−0.655 **	0.766 **	1

Note: ** Correlation is significant at the 0.01 level (2-tailed); PDI: privacy disclosure intention PR: perceived risk; PB: perceived benefits; PRE: perceived relevance.

**Table 8:** Mediation analyses.

Predictors	Model 1 (PR)			Model 2 (PB)			Model 3 (PDI)		
	b	SE	t	b	SE	t	b	SE	t
Constant	7.274	0.279	26.026 ***	1.675	0.219	7.667 ***	3.808	0.484	7.864 ***
PRE	−0.615	0.041	−14.878 ***	0.661	0.032	20.440 ***	0.298	0.051	5.786 ***
Gender	−0.259	0.141	−1.823	−0.082	0.111	−0.740	0.013	0.110	0.118
Age	−0.003	0.050	−0.053	−0.072	0.038	−1.851	0.007	0.044	0.192
Educational level	0.067	0.130	0.516	−0.157	0.102	−1.539	−0.060	0.101	−0.590
PR							−0.513	0.051	−10.023 ***
PB							0.322	0.066	4.911 ***
R^2^	0.661			0.771			0.869		
*F*-Value	55.850 ***			105.392 ***			146.677 ***		

Note: *** Correlation is significant at *p* < 0.001 (2-tailed); PDI: privacy disclosure intention; PR: perceived risk; PB: perceived benefits; PRE: perceived relevance.

**Table 9:** Test of the moderated mediational model.

DV	IV	b	SE	t	LLCI	ULCI	R^2^	*F*-Value
PDI	Constant	5.105	0.435	11.746 ***	4.250	5.961	0.869	295.839 ***
	PRE	0.296	0.051	5.816 ***	0.196	0.396		
	PR	−0.514	0.051	−10.160 ***	−0.614	−0.415		
	PB	0.323	0.065	5.005 ***	0.196	0.449		
PR	Constant	4.394	0.097	45.421 ***	4.204	4.584	0.659	74.004 ***
	PRE	−0.634	0.061	−10.322 ***	−0.755	−0.513		
	Temporal	0.195	0.136	1.432	−0.081	0.463		
	ARE × Temporal	0.051	0.083	0.614	−0.092	0.216		
PB	Constant	4.604	0.072	63.790 ***	4.462	4.746	0.793	163.291 ***
	PRE	0.568	0.046	12.388 ***	0.477	0.658		
	Temporal	−0.546	0.101	−5.377 ***	−0.746	−0.346		
	ARE × Temporal	0.136	0.062	2.184 *	0.013	0.258		

Note: * *p* < 0.05, *** *p* < 0.001 (2-tailed); Bootstrap sample size = 5000; DV: dependent variable; IV: independent variable; ULCI: upper limit of confidence interval; LLCI: lower limit of confidence interval.

**Table 10:** Conditional indirect effects of PB on PDI according to values of the moderator.

Values of Moderators (Temporal)	Indirect Effect	SE	LLCI	ULCI
0	0.183	0.039	0.110	0.265
1	0.227	0.048	0.138	0.324
Index of moderated mediation	0.044	0.023	0.001	0.091

Note: 0 = near; 1 = distant.

## Data Availability

The data used to support the findings of this study are available from the corresponding author upon request.

## Appendix A

**Table A1:** Questionnaire.

Variable	Questions	References
Privacy Disclosure Intention (PDI)	Q1: I am willing to authorize or provide access to my geolocation (or list of applications)	(Li et al., 2010; Liu et al., 2022)
	Q2: I do not perceive any concerns regarding authorizing this application to access my geolocation (or list of applications)	
	Q3: I regard it as reasonable and appropriate to authorize or provide access to my geolocation (or list of applications)	
Perceived Risk (PR)	Q4: I perceive a high level of risk in authorizing this application to access my geolocation (or list of applications)	(Zhou, 2011)
	Q5: I would remain highly vigilant if this application requests access to my geolocation (or list of applications)	
	Q6: I am concerned that authorizing or providing this application with access to my geolocation (or list of applications) could lead to improper use	
	Q7: I am worried that authorizing or providing this application with access to my geolocation (or list of applications) may result in potential losses	
Perceived Benefits (PB)	Q8: Providing my geolocation (or list of applications) to this application could bring certain benefits	(Sun et al., 2014; T. Wang et al., 2016)
	Q9: Sharing geolocation information (or list of applications) with this application could enhance my shopping experience	
	Q10: Authorizing this application to access my geolocation (or list of applications) could enable me to enjoy better services	
Perceived Relevance (PRE)	Q11: The request for geolocation (or list of applications) by this online shopping app is directly related to the shopping services it provides	(Hajli & Lin, 2016)
	Q12: The online shopping app’s request for geolocation (or list of applications) is reasonable, as it facilitates the provision of shopping services	
